# Identification of Biomarkers Related to Liquid-Liquid Phase Separation for Ulcerative Colitis Based on Single-Cell and Bulk RNA Transcriptome Sequencing Data

**DOI:** 10.2174/0118715303355042241208171133

**Published:** 2025-01-08

**Authors:** Jicheng Lu, Xu Lu, Bin Chen

**Affiliations:** 1 Department of Oncology, Suzhou Ninth People’s Hospital, Suzhou, 215200, China;; 2 Department of Radiotherapy, Suzhou Ninth People’s Hospital, Suzhou, 215200, China

**Keywords:** LLPS, inflammatory bowel disease, ulcerative colitis, single-cell sequencing analysis, gene set enrichment analysis (GSEA), biomarkers

## Abstract

**Background:**

Liquid-Liquid Phase Separation (LLPS) is a process involved in the formation of established organelles and various condensates that lack membranes; however, the relationship between LLPS and Ulcerative Colitis (UC) remains unclear.

**Aims:**

This study aimed to comprehensively clarify the correlation between ulcerative colitis (UC) and liquid-liquid phase separation (LLPS).

**Objectives:**

In this study, bioinformatics analyses and public databases were applied to screen and validate key genes associated with LLPS in UC. Furthermore, the roles of these key genes in UC were comprehensively analyzed.

**Methods:**

Based on the single-cell transcriptomic data of UC obtained from the Gene Expression Omnibus (GEO) database, differences between patients with UC and their controls were compared using the limma package. The single-cell data were then filtered and normalized by the ‘Seurat’ package and subjected to dimension reduction by the Uniform Manifold Approximation and Projection (UMAP) algorithm. The LLPS-related genes (LLPSRGs) were searched on the DrLLPS website to obtain cross-correlated genes, which were scored using the ssGSEA algorithm. Next, functional enrichment, interaction network, immune landscape, and diagnostic and drug prediction of the LLPSRGs were comprehensively explored. Finally, the results were validated using external datasets and quantitative real-time PCR (qRT-PCR).

**Results:**

A total of eight cell types in UC were classified, namely, fibroblasts, macrophages, endothelial cells, neutrophils, NK cells, B cells, epithelial cells, and T cells. The intersection between differently expressed genes (DEGs) among the eight cell types identified 44 key genes, which were predominantly enriched in immune- and infection-related pathways. According to receiver operating characteristic (ROC) curves, *PLA2G2A*, *GZMK*, *CD69*, *HSP90B1*, and *S100A11* reached an AUC value of 0.94, 0.95, 0.86, 0.89, and 0.93, respectively. Drug prediction revealed that decitabine, tetrachlorodibenzodioxin, tetradecanoylphorbol acetate, thapsigargin, and cisplatin were the potential small molecular compounds for *PLA2G2A*, *GZMK*, *CD69*, *HSP90B1*, and *S100A11*. Immune cell infiltration analysis demonstrated that the infiltration of CD4 memory T cell activation, macrophage M1, T macrophage M0, neutrophils, and mast cell activation was higher in the UC group than in the normal group.

**Conclusion:**

The LLPSRGs play crucial roles in UC and can be used as prognostic and diagnostic markers for UC. The current findings contribute to the management of UC.

## 1 INTRODUCTION

Inflammatory Bowel Disease (IBD) is divided into Crohn's disease (CD) and ulcerative colitis (UC). The causes of IBD include genetic susceptibility, changes in gut microbial environmental factors, and disrupted mucosal immune homeostasis. In general, a normal colon interacts with a variety of bacteria. However, chronic inflammation, damage, and disability of the gastrointestinal (GI) tract will occur when such a microscopic balance is disturbed. UC is characterized by abdominal pain, recurrent diarrhea, and mucopurulent blood in the stool [1, 2]. Currently, though endoscopy is the primary diagnostic method of UC, it sometimes fails to detect early lesions. Previous studies have discovered several biomarkers to indicate inflammatory changes, but these biomarkers are less accurate in identifying disease-related details, such as cell-to-cell interactions, cell-type specific expression, and cell fraction. Therefore, screening effective biomarkers for improving early diagnosis and effective treatment for UC is of great importance.

Various structures without membranes, such as nucleoli, promyelocytic leukemia (PML) nuclear bodies (PML-NBs), and P bodies in *C. elegans*, are present in eukaryotic cells and play critical functions [3, 4]. Recent studies indicated that these structures are formed based on the principle of LLPS, which reclassifies these structures as “membrane-less condensates” or “biological condensates” [5]. Numerous vital biological processes, including X-chromosome inactivation, chromatin organization, transcription, responses to DNA damage, tumor development, and autophagy, employ LLPS to create the corresponding membrane-less condensates and fulfill their own functions [6, 7]. Moreover, LLPS also contributes to the development of inflammatory and neurodegenerative diseases and cancers [8-24]. Currently, the role of LLPS in the development and progression of UC remains unclear, pointing to an urgent need to discover LLPS-related markers to provide novel possibilities for the management of UC.

In recent years, scRNA-seq has emerged as a groundbreaking technique for exploring the transcriptomes of various cell types [25, 26]. ScRNA-seq characterizes the overall gene expression profiles of individual cells by employing advanced next-generation sequencing technologies, thereby enabling the exploration of heterogeneity within cellular populations [27, 28]. Combining scRNA-seq with bulk RNA sequencing, new biomarkers for cancers have been increasingly discovered [29-31]. For example, Jiang *et al.* constructed and validated a prognostic model for LUAD by integrating 10 × scRNA-seq and bulk RNA-seq data and observed two distinct subtypes with different prognostic and immune characteristics in this population [29]. He *et al.* identified the CREM gene based on scRNA-seq and bulk data and used *in vitro* assays to verify that it is highly expressed in both lesion-free and lesion-site colonic mucosa of UC patients [30]. Similarly, Dai *et al.* also analyzed peripheral blood cell subtypes of UC patients by scRNA-seq and combined with bulk RNA-seq, which, in turn, revealed the core genes of UC [31]. Thus, this study performed an integrated analysis with a focus on mining effective diagnostic biomarkers for UC and also investigated the role of LLPS in UC. We identified biological pathways predominantly expressed in immune cells and used two machine-learning methods to filter characteristic genes involved in UC. Statistically significant results were obtained and validated in the external dataset. The current findings will provide potential key markers and therapeutic targets for the diagnosis and treatment of UC.

## 2 MATERIALS AND METHODS

### 2.1 Acquisition of Transcriptome Data

 The transcriptome data used in this study were obtained from GEO. The GSE875466 dataset was utilized to identify DEGs between UC patients and healthy controls. The single-cell dataset GSE214695 was used for single-cell analysis. Additionally, GSE36807 was used to confirm the expression of the biomarkers selected by a series of computational methods.

### 2.2 Acquisition of DEGs

Differential genes between UC patients and normal controls were selected by performing differential analysis using the limma package in the R software (version 3.40.6).

### 2.3 Acquisition and Processing of the scRNA-seq Data

The single-cell sequencing dataset containing six UC patients and six healthy controls was downloaded from the GEO database. First, the R package “Seurat” was utilized to filter the single-cell sequencing data, and the expression data was normalized with a linear regression model to obtain key cells. Next, data from variance analysis on the key cells were subjected to the canonical correlation analysis (CCA) method in the “Seurat” to eliminate the batch effect. Next, the Uniform Manifold Approximation and Projection (UMAP) algorithm was applied to reduce dimensionality, followed by performing clustering analysis on the top 20 principal components (PCs). Lastly, the R package “singleR” and the CellMarker database were utilized to mine differential cell marker genes for cell annotation. Subsequently, potential marker genes of each cell type were screened from the annotated cells using the “FindAllMarkers” function, with HumanPrimaryCellAtlasData, BlueprintEncodeData, and ImmuneCellExpressionData as reference data for auxiliary annotation. Significant differential maker genes (DMGs) in each cell type were identified by the Wilcoxon rank-sum test.

### 2.4 Screening of LLPSRGs

We screened a total of 3598 LLPSRGs from the DrLLPS website (http://llps.biocuckoo.cn/) for subsequent research.

### 2.5 Single Sample GSEA (ssGSEA) Analysis

The scores of LLPSRGs in the annotated cells of all the samples were calculated by the ssGSEA algorithm in the “GSVA” R package.

### 2.6 Identification of Intersection Genes and Functional Enrichment Analysis

The FindMarkers function in the R package “Seurat” was applied to compare the differences between the sequencing data of two groups of cells (high-LLPSRG correlation score group and low-LLPSRG correlation score group) and to select the DEGs between the two groups. The DEGs were then intersected with the DMGs to obtain cross-correlated genes.

To predict the functions of these genes, GO and KEGG pathway analyses were conducted by applying the R package clusterProfiler. Statistical significance was considered when *p*-value < 0.05.

### 2.7 Protein-Protein Interaction (PPI) Network Establishment

The PPI network of the 44 intersecting genes was developed using the STRING online tool with a combined score of  > 0.4 as the screening criterion, while disconnected nodes were hidden in the network.

### 2.8 Least Absolute Shrinkage And Selection Operator (LASSO) Analysis

The R software “glmnet” package was employed to perform LASSO logistic regression analysis based on the intersection of genes in 87 disease groups and 21 healthy control groups.

### 2.9 Support Vector Machine Recursive Feature Elimination (SVM-RFE)

SVM-RFE is an algorithm that iteratively removes features with the lowest weights in the SVM model, and then the remaining features are used to train the model again. The desired number of features is chosen by the second iteration.

### 2.10 ROC Analysis

The validity of the model was assessed using ROC to calculate the AUC of the model.

### 2.11 Gene Set Enrichment Analysis (GSEA)

The samples were classified into two groups based on the expression levels of the five hub genes by performing GSEA (http://software.broadinstitute.org/gsea/index.jsp) in the GSEA software (version 3.0), and relevant pathways and molecular mechanisms were analyzed using the c2. Cp. Kegg. V7.4.gmt subset from Molecular Signatures Database MSigDB, http://www.gsea-msigdb.org/gsea/downloads.jsp). The gene set of a minimum of 5 genes to a maximum of 5000 genes was subjected to 1000 iterations, according to gene expression profiles and phenotypic grouping. Statistically significant was defined when the *p-*value was <0.05 and FDR<0.25.

### 2.12 Drug Prediction

The gene-chemical interactions of the five hub genes were searched in the CTD database (https://ctdbase.org/), and the compounds were ranked according to their interactions.

### 2.13 Immune Infiltration Landscape

The expression data of the UC samples and normal samples were loaded into CIBERSORT (https://cibersort.stanford.edu/), and the algorithm was repeated 1000 times to quantify and compare the relative proportion of 22 immune cell types [32] in the two groups. The results were visualized on a landscape map.

### 2.14 Quantitative Real-Time PCR (qRT-PCR) and Validation in an External Dataset

Surgical samples from the colon were collected from 10 UC patients and 5 healthy donors. The RNA levels were measured by RT-PCR. MRNA was extracted by Trizol Reagent (Thermo Fischer Scientific) and reverse-transcribed by Evo M-MLV RT Master Mix (Accurate Biology) for quantitative PCR (qPCR). QRT-PCR was performed on an ABI 7500 real-time PCR thermocycler. After normalization for β-actin, the relative proportion of target transcripts from duplicate samples was calculated. Transcriptome levels were determined, and the differences were compared using the Wilcoxon test. The primer sequences are shown in Table **S1**. Finally, the expressions of the five genes were validated using the external dataset GSE36807.

## 3 RESULTS

### 3.1 Acquisition of DEGs

**Fig. (1):**
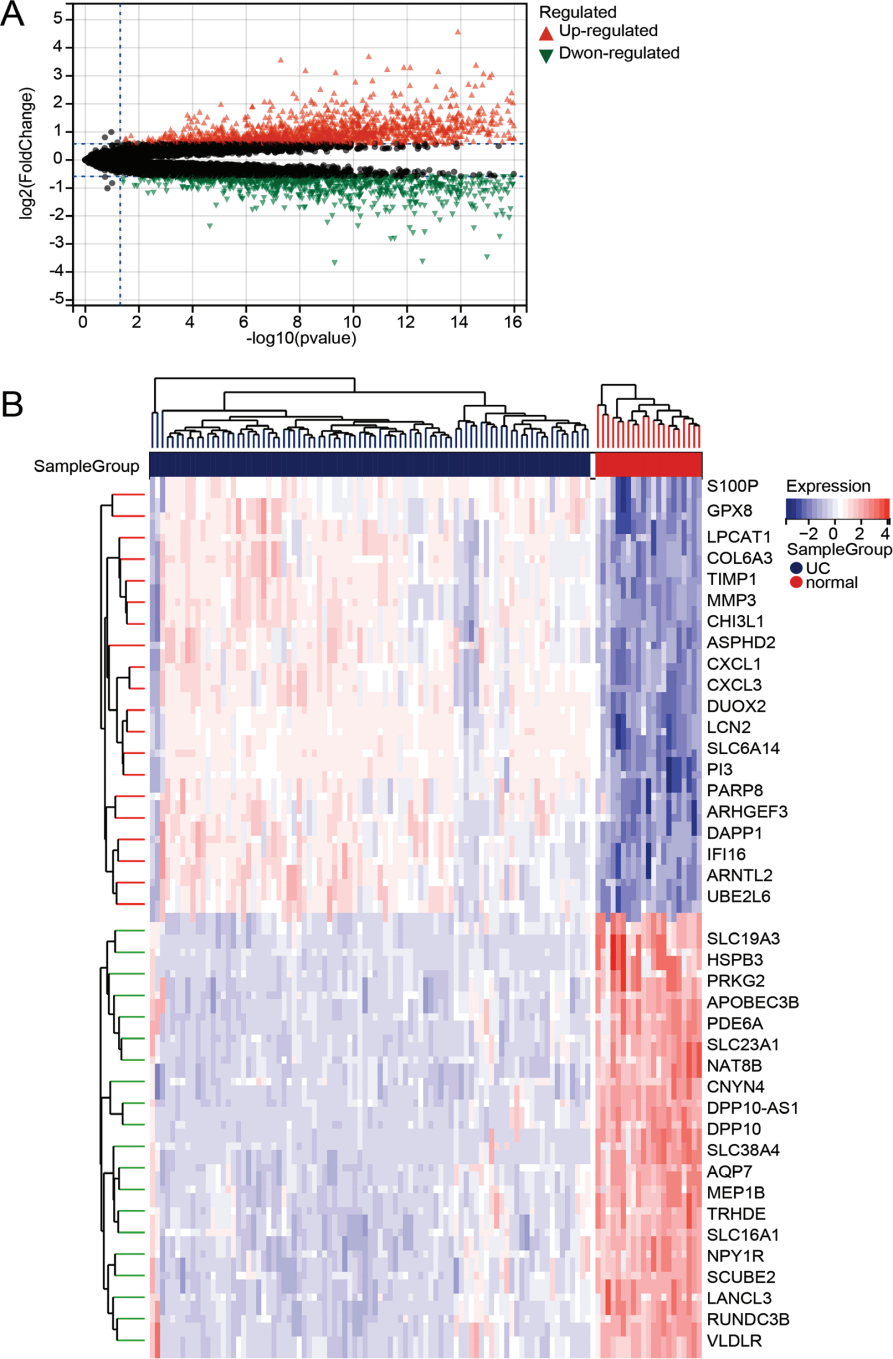
Volcano map (**A**) and heat map (**B**) of upregulated and downregulated genes.

Analysis of the GSE875466 dataset identified 1000 down-regulated genes and 1474 up-regulated genes (Fig. **1**).

### 3.2 Single-Cell Sequencing Analysis

**Fig. (2):**
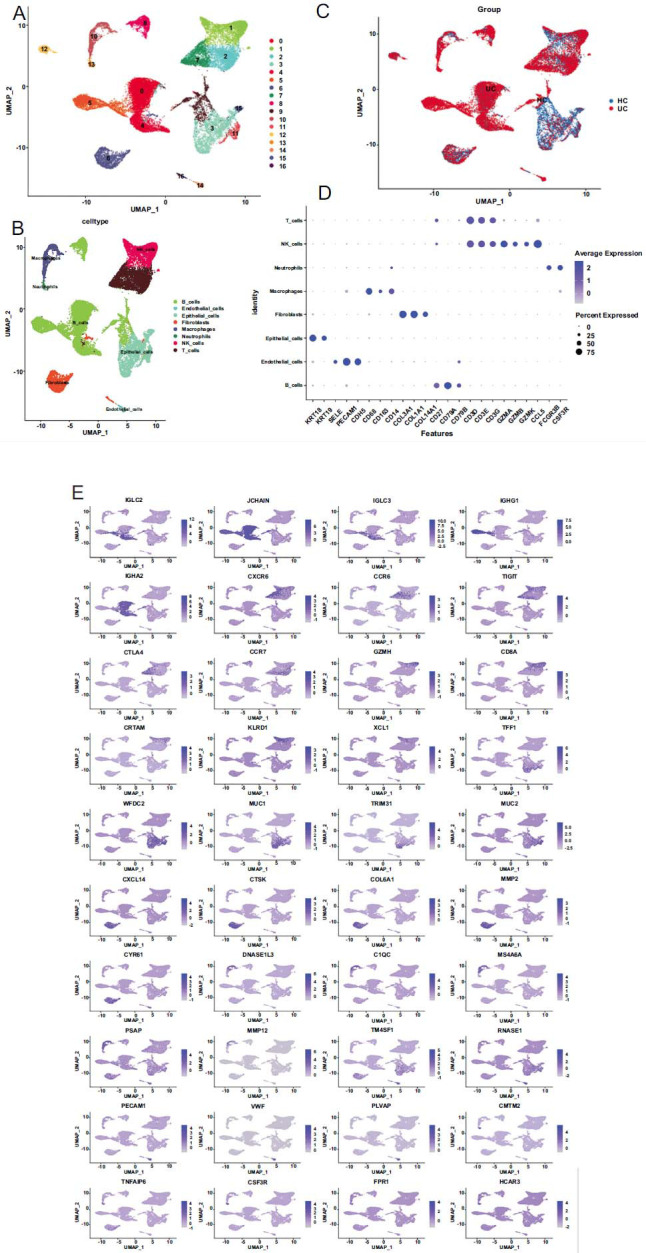
Single-cell analysis. (**A**). Clustering results. (**B**). The resulting annotation. (**C**). The clustering results of different groups. (**D**). Bubble plot of potential marker genes. (**E**). The feature plots for the top genes for all subpopulations.

After dimensionality reduction by UMAP, the single-cell sequencing datasets were clustered and annotated using SingleR. The results showed that the cells mainly belonged to eight primary cell types, which were subsequently assigned into 17 subgroups (subgroup 0 to 16) (Figs. **2A** and **2B**). The single-cell data for the normal and UC samples are displayed in Fig. (**2C**). After removing duplicates, a total of 875 potential marker genes were obtained, and five marker genes in each cluster were visualized in the bubble plot in (Fig. **2D**). Fig. (**2E**) lists the top genes in all the subpopulations.

**Fig. (3):**
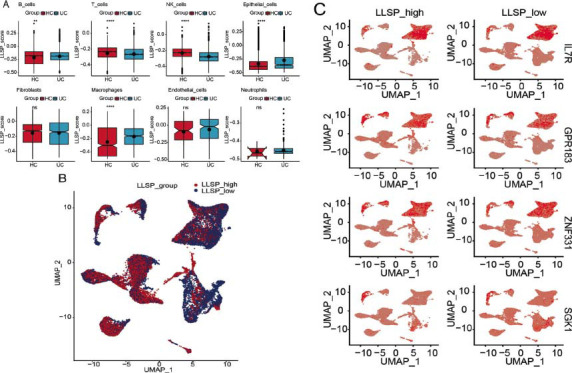
(**A**): Differential analysis of phase separation correlation scores in disease and controls. (**B**): High and low grouping separate cells. (**C**): The most obvious differences in macrophages 4 genes.

LLPS-related scores for different cells in all the samples were calculated using the ssGSEA algorithm (Fig. **3A**). As the LLPS-related scores of epithelial_cells, NK_cells, B_cells, T_cells, and macrophages were noticeably different between disease and control groups, these five cells were therefore considered as key cells. Subsequently, all the cells were divided into a high-LLPS correlation score group and a low-LLPS correlation score group based on the median LLPS correlation score (-0.2297). In Fig. (**3B**), red indicates the distribution of cells in the high-LLPS correlation score group. Subsequently, 289 DEGs between the two groups were identified by the FindMarkers function, and the four most significant DEGs in macrophages are shown in Fig. (**3C**).

### 3.3 Screening Intersection Genes and Functional Assessment and Machine Learning to Select UC Biomarkers

**Fig. (4):**
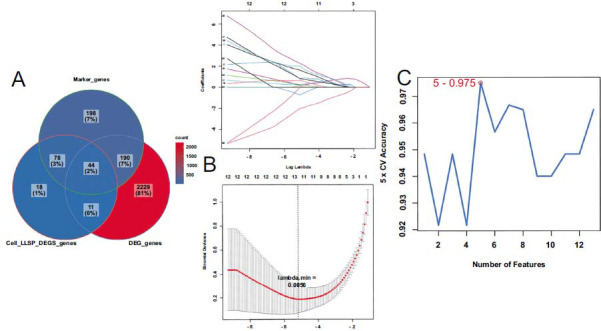
(**A**): Intersection gene screening. (**B**): LASSO. (**C**): SVM - RFE.

**Fig. (5):**
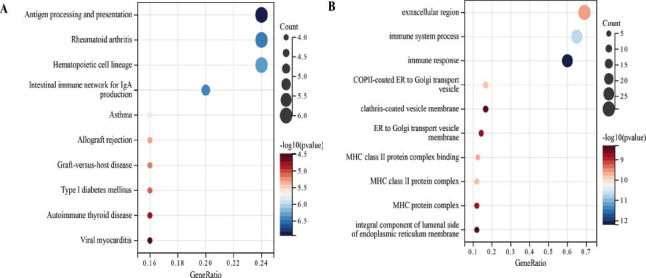
Enrichment analysis of 44 intersection genes (**A**): KEGG (**B**): GO.

A sum of 44 differential maker genes (DMGs) in the intersection of NK cells, T cells, epithelial cells, B cells, macrophages, LLPS differential genes, and bulk-RNA seq DEGs were identified in Fig. (**4A**). GO analysis showed that these genes were largely enriched in the physiological processes, including the immune system process, MHC protein complex, clathrin-coated vesicle membrane, integral component of lumenal side of endoplasmic reticulum membrane, ER to Golgi transport vesicle membrane, extracellular region, MHC class II protein complex binding, COPII-coated ER to Golgi transport vesicle, MHC class II protein complex, and immune response (Fig. **4**). KEGG pathway analysis demonstrated that these genes were enriched in pathways involved in various physiological and pathological processes, including rheumatoid arthritis, autoimmune thyroid disease, hematopoietic cell lineage, type I diabetes mellitus, antigen processing and presentation, graft-versus-host disease, intestinal immune network for IgA production, asthma, viral myocarditis, hematopoietic cell lineage, allograft rejection. The top 10 GO terms and KEGG pathways were visualized by the R package “clusterProfiler” according to the *p*-value (Fig. **5**).

Based on the 44 intersection genes, 13 genes (*PLA2G2A, GZMK, CD69, HSP90B1, S100A11, ENO1, ACAA2, CD74, SPINK4, LCN2, OLFM4, MGST1,* and *HLA-DPA1*) were selected from 87 disease groups and 21 control groups by LASSO logistic regression in Fig. (**4B**). Then, SVM-RFE machine learning with 5-fold cross-validation further determined 5 genes (*PLA2G2A, GZMK, CD69, HSP90B1,* and *S100A11*) as the biomarkers for UC in Fig. (**4C**).

### 3.4 Verification of the Expressions of the Five Hub Genes in UC Tissues

**Fig. (6):**
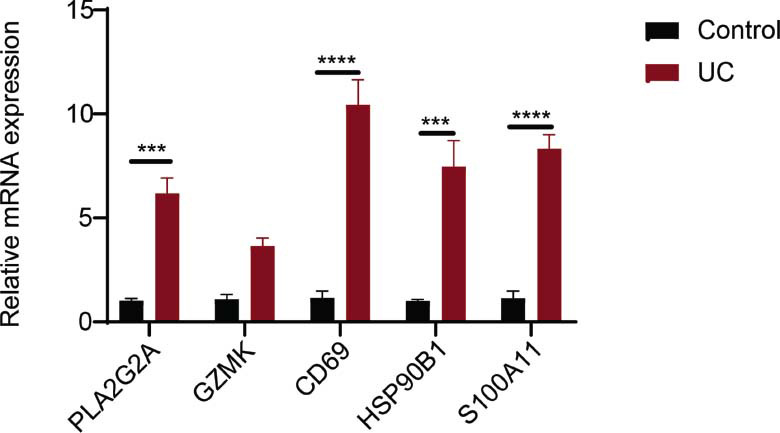
PCR validation of KEY gene expression.

Detection of the expressions of *PLA2G2A, GZMK, CD69, HSP90B1,* and *S100A11* in UC and normal tissues by qPCR showed that the expressions of *PLA2G2A, CD69, HSP90B1,* and *S100A11* were significantly higher in the UC tissues, which was in accordance with the validation results from an external dataset. The expression level of *GZMK* exhibited no statistically significant difference between the two types of tissues, but it demonstrated an upward trend (Figs. **5** and **6**).

### 3.5 PPI Networks

Using the STRING online tool, we developed a PPI network containing 103 edges and 33 nodes to reflect the interrelationships of the 44 intersecting genes in Fig. (**7**).

### 3.6 ROC Curve Analysis

The five diagnostic markers were combined into a model using OpenGL Mathematics (GLM) in Fig. (**8A**). All five markers had an AUC value greater than 0.8, and the combination of the five genes reached an AUC of 1, indicating an excellent performance of the model. The five genes were significantly high-expressed in the UC group and low-expressed in the control group in Fig. (**8B**).

### 3.7 Gene Set Enrichment Analysis (GSEA)

Activated signaling pathways that expressed the five genes in high-LLPS and low-LLPS correlation score groups were identified and compared using GSEA. Gene sets differently enriched in the two groups were associated with the synthesis and metabolism of cellular components, immune-related pathways, infection-related pathways, such as arginine and proline metabolism, glycerophospholipid metabolism, T cell receptor signaling pathways, cytokine receptor interactions, leishmaniasis infection, and pathogenic Escherichia coli infection in Figs. (**8C**-**G**).

### 3.8 Evaluation of the Potential Therapeutic Agents

Potential drugs for each gene and the drugs most actively interacting with the five genes were identified. The PubChem website was used to visualize the five small molecular compounds in Fig. (**9**). The current results indicated that these small molecular compounds were potential agents for UC treatment.

### 3.9 Analysis of Immune Cell Infiltration

The CIBERSORT algorithm was utilized to quantify 22 subtypes of immune cells in UC patients with a *p*-value of < 0.05 as a cut-off. It was found that the infiltration of macrophage M0, T cell CD4 memory activation, mast cell activation, neutrophils, and macrophage M1 were higher in the UC group than in the normal group, while that of mast cells resting, NK cells activated, dendritic cells resting, T cells regulatory (Tregs), and macrophages M2 were higher in the normal group than the UC group in Fig. (**10**).

## 4 DISCUSSION

This study explored the clinical significance of LLPS in UC by performing bioinformatics analysis. A total of 44 significantly differentially expressed LLPSRGs were obtained based on the data of bulk RNA-seq and scRNA-seq of UC patients from the GEO database. A total of 5 genes (*PLA2G2A, GZMK, CD69, HSP90B1*, and *S100A11*) out of the 44 genes were determined as the biomarkers for UC by LASSO regression analysis and SVM-RFE. *PLA2G2A* is characterized as an acute-phase protein under the transcriptional control of pro-inflammatory cytokine signaling [33]. Previous studies found that the expressions of pro-inflammatory genes, including *PLA2G2A,* are upregulated in trinitrobenzene sulfonic acid-induced murine colitis [34]. As a member of the serine-proteases family, age-associated granzyme K (*GZMK)* is mainly expressed by T lymphocytes. Denis *et al.* identified *GZMK*-expressing CD8+ T (Taa) cells as a potential target for age-related dysfunction of the immune system [35]. *CD69* is the earliest inducible surface antigen expressed by activated lymphocytes and is expressed on almost all blood cells after induction, such as NK cells, monocytes, T cells, neutrophils, B cells, eosinophils, thymocytes, *etc.* *CD69* is expressed only after cell activation; for this reason, it has now become a marker molecule for cell activation [36-38]. Hu *et al.* considered CD69 to be one of the hub genes associated with immune-infiltrated cells in UC [39]. Compared with healthy controls, Haga *et al.* found that the frequency of mucosal-correlated invariant T cells was significantly decreased in the peripheral blood derived from patients with UC. The expression level of *CD69* in these cells is indicative of endoscopic score, plasma IL-18 level, and disease activity [40]. *HSP90b1* is a main downstream chaperone in the endoplasmic reticulum (ER) that mediates the ER unfolded protein response (UPR), which is crucial in both early B-cell development and plasma cell differentiation [41]. Wang *et al.* found obvious acetylation of lysine 142 in *HSP90B1* in the UC colon and that the secretion of TNF-α and IL-2 is reduced by the point mutation of HSP90B1-K142ac in LPS-stimulated cultured cells [42]. *S100A11* belongs to the S100 family and acts as a calcium sensor/binding protein to fulfill its biological functions inside and outside the cell. *S100A11* plays a crucial role in diseases, including cancers, metabolic diseases, and inflammatory diseases [43-46]. Zhang *et al.* revealed that nine co-expressed genes, including *S100A11,* are associated with neutrophil infiltration-mediated inflammation and immune regulation in IBD patients [47].

Previous studies have confirmed that the above five genes can promote the occurrence and progression of UC by affecting immune regulation and inducing inflammatory response, suggesting that these 5 genes could be the targets for UC treatment. Therefore, we screened five potential small molecular compounds for each gene, including decitabine, tetrachlorodibenzodioxin, tetradecanoylphorbol acetate, thapsigargin, and cisplatin. Su *et al.* suggested that decitabine causes the release of inhibitory factor IL 10, reduction of pro-inflammatory factor IL 17, activation of CD^4+^ Foxp^3+^ T cells, and inhibition of MAPK pathway activation to alleviate DSS-impaired colon barrier, mucus, and bloody stools as well as weight loss in the mice [48]. Tetrachlorodibenzodioxin, also known as 2,3,7,8-tetrachlorodibenzo-p-dioxin (TCDD), is a potent aryl hydrocarbon receptor (AhR) activator. The AhR pathway plays a variety of roles in cellular functions and is involved in the immune system [49-51]. Takamura *et al.* confirmed that activation of the AhR pathway by TCDD could ameliorate DSS-induced colitis in mice, part of which was at least achieved by PGE2 production [52]. In addition, acetylcholinesterase (AChE)-targeting miR-132 induced by TCDD could enhance cholinergic anti-inflammation [53]. Tetradecanoylphorbol acetate (TPA), alternatively known as phorbol-12-myristate-13-acetate (PMA), is the most used phorbol ester. TPA binds to and activates protein kinase C, exerting wide effects on cells and tissues [54, 55]. Li *et al.* found that PMA significantly blocks the protective activity of dihydroartemisinin against colitis *in vitro* [56]. Thapsigargin (TG) is derived from *Thapsia garganica* and functions as a promoter of tumor cells in mammalian cells. TG can specifically inhibit endoplasmic reticulum Ca^2+^-ATPase and discharge intracellular Ca^2+^ stores, thereby rapidly increasing cytosolic Ca^2+^ concentration [57, 58]. TG is used to induce endoplasmic reticulum stress (ERS) [59-61]. Therefore, TPA and TG might not be useful for UC treatment. Cisplatin is an antineoplastic chemotherapy agent that realizes its effect through cross-linking with DNA to cause DNA damage in tumor cells [62-64]. However, the therapeutic roles of cisplatin in UC have not been studied. Some previous reports showed that cisplatin could induce autophagy by activating some key pathways [65] and that autophagy plays an essential role in regulating intestinal immunity by maintaining the balance between commensal flora and intestinal epithelial cells [66], suggesting that cisplatin might be effective in treating UC.

Whether UC is caused by immune dysregulation as a result of genetic abnormalities and environmental factors remains unclear. However, studies have found that one of the important pathological features of UC is the infiltration of inflammatory cells. This study used single-cell analysis combined with the ssGSEA algorithm to calculate the LLPS correlation scores for different cells in all the samples and identified five cells (macrophages, epithelial cells, T cells, NK cells, and B cells) as the key cells for further analysis. T cells, especially Treg and Th17 cells, play a crucial role in intestinal immunity as they can release inflammatory cytokines through different transcriptional pathways to regulate intestinal immunity, but Treg and Th17 cells function oppositely. Specifically, Treg inhibits immune response and inflammatory process and regulates immune response and autoimmune tolerance. A comparison of the difference in immune cell infiltration between the UC group and the normal control group using the CIBERSORT algorithm demonstrated that the proportion of Treg cells was lower in the UC group, suggesting that regulating Treg/Th1h immune balance and increasing the number of Treg cells can reduce intestinal inflammation. A previous study analyzed the role of Treg and Th17 cells in bowl diseases with traditional Chinese medicine [67-69]. In addition, phase separation and T-cell function are inextricably linked. Linker for activation of T-cells (LAT) is an intermediate component of the T-cell receptor (TCR) signaling cascade that connects the upstream TCR activation *via* antigen presentation with downstream effects, including Erk signaling and actin polymerization, which are two processes for triggering proper T-cell response [70]. Using *in vitro* reconstitution, Su *et al.* demonstrated that LAT signaling clusters arise through LLPS *via* multivalent interactions among Sos1, phosphorylated LAT (pLAT), and Grb2 [8]. These liquid-like structures could recruit pathway activators and selectively exclude repressors, such as phosphatases, greatly promoting TCR downstream signaling by forming a signaling compartment. This indicated that induction of LLPS could modulate T-cell function in UC. Macrophages also have bidirectional regulatory effects on immune responses. Activated M1 macrophages tend to cause chronic inflammation and tissue damage, while M2 macrophages are alternatively activated and could eliminate inflammatory responses and promote wound healing. The two activated forms of macrophages function together to maintain the immune balance in the body, while abnormal immune regulation of either one may destroy immune homeostasis and cause related diseases [71]. Our analysis also showed that the proportion of macrophages M1 was higher, and that of M2 was lower in the UC group. Some studies have demonstrated that targeting macrophages can alleviate intestinal inflammation by changing the polarity of macrophages from M1 to M2 in the colon [72-74]. Sun *et al.* revealed that macrophage reprogramming driven by LLPS may be the mechanism underlying pathogen-induced inflammatory progression in UC [75].

It should be equally noted that our study still had some limitations. Firstly, due to the small sample size in our study, more patients of different age groups, genders, and disease courses are required to be included in future studies to improve the generalizability of the results. In addition, *in vivo* and *in vitro* models will be used to perform functional experiments on the key genes discovered in this research. Finally, although the expression patterns of LLPS-related genes in UC have been analyzed, the specific mechanisms of LLPS in UC still remain to be explored in depth. Subsequent studies will further investigate how LLPS affects cellular functions in the pathogenesis of UC by integrating multi-omics data and analyzing the regulatory mechanisms of LLPS in different cell types.

## CONCLUSION

Combining single-cell analysis and bulk RNA sequencing data, this study screened five genes (*PLA2G2A*, *GZMK*, *CD69*, *HSP90B1*, and *S100A11*) associated with LLPS as the biomarkers for UC using bioinformatics methods and showed a significant diagnostic value of all the five genes in UC. These markers were involved in cellular components, immune-related pathways, and infection-related pathways in UC. Considering that LLPS and UC were related to the cellular immune microenvironment, we further discussed the relationship between immune cell infiltration and these biomarkers and found that LLPS caused immune dysfunction to trigger the development of UC. In conclusion, our study provides a new direction for the early diagnosis and individualized treatment of UC and offers five core genes as novel molecular markers for UC.

## AUTHORS’ CONTRIBUTIONS

The authors confirm their ciontribution to the paper as follows: J.C.L., X.L., and B.C. contributed to the concept and design of the study. X.L. provided the technical support. J.C.L. proposed the methodology and conducted the experiment. J.C.L. and X.L. took part in the acquisition, analysis, or interpretation of the data. X.L. curated the software and performed statistical analysis. J.C.L. designed the pictures and tables. B.C. drafted the manuscript, modified the language, and provided guidance. All authors approved the final version of the manuscript for submission.

## Figures and Tables

**Fig. (7):**
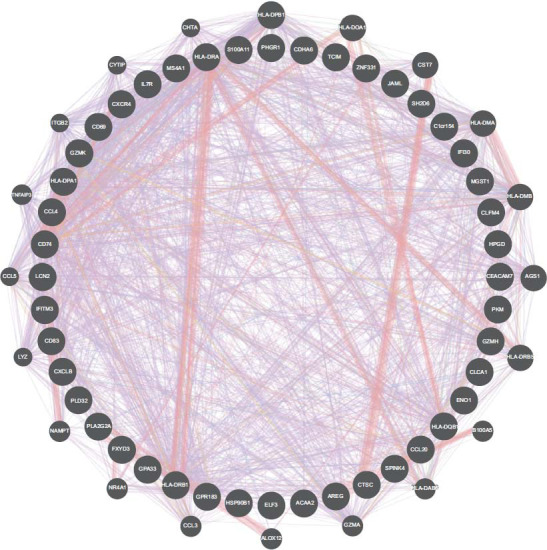
PPI network.

**Fig. (8):**
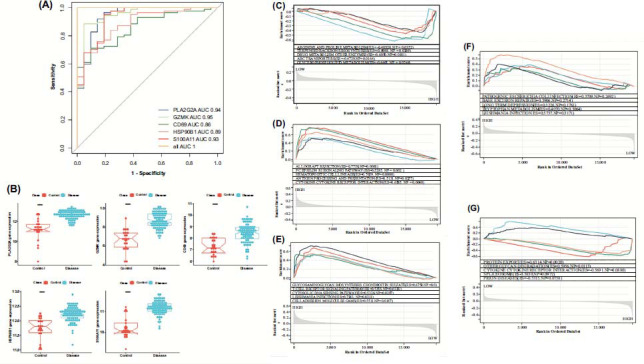
(**A**): ROC curve. (**B**): Expression of the five genes in the disease and control groups. (**C**-**G**): *GSEA*. (**C**): *PLA2G2A* (**D**): *GZMK* (**E**): *CD69* (**F**): *HSP90B1* (**G**): *S100A11*.

**Fig. (9):**
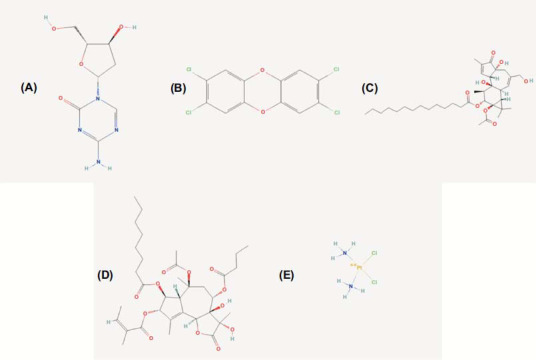
(**A-E**) Five small molecular compounds may be potential UC treatment.

**Fig. (10):**
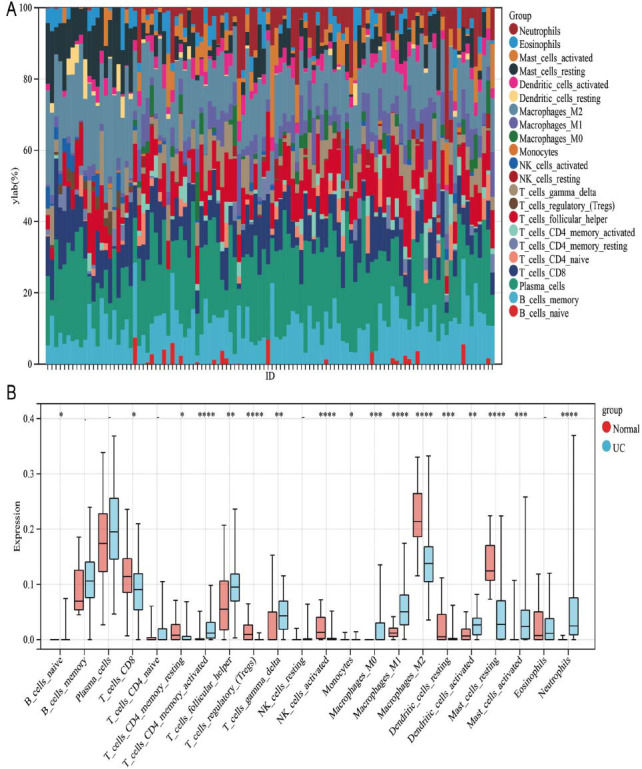
Analysis of immune cell infiltration. (**A**): The proportion of 22 subtypes of immune cells in UC patients was calculated using the CIBERSORT algorithm. (**B**): Boxplot was used to compare the differences in cell infiltration abundance between the two subgroups using the CIBERSORT algorithm.

## Data Availability

The datasets generated and/or analyzed during the current study are available in the (GSE875466) repository, (https://www.ncbi.nlm.nih.gov/geo/query/acc.cgi?acc=GSE875466), (GSE214695) repository, (https://www.ncbi.nlm.nih.gov/geo/query/acc.cgi?acc=GS214695) and (GSE36807) repository, (https://www.ncbi.nlm.nih.gov/geo/query/acc.cgi?acc=GSE36807).

## LIST OF ABBREVIATIONS

IBD: Inflammatory Bowel Disease
UC: Ulcerative Colitis
LLPS: Liquid-Liquid Phase Separation
GEO: Gene Expression Omnibus
CCA: Canonical Correlation Analysis
DEGs: Differentially Expressed Genes
LLPSRGs: LLPS-Related Genes
CD: Crohn's Disease
GI: Gastrointestinal
LAT: Linker for Activation of T-Cells
LLPS: Liquid-Liquid Phase Separation
qRT-PCR: Quantitative Real-Time PCR
TCR: T-Cell Receptor
TCDD: Tetrachlorodibenzo-p-Dioxin
TG: Thapsigargin
TPA: Tetradecanoylphorbol Acetate
UC: Ulcerative Colitis
UMAP: Uniform Manifold Approximation and Projection
DMGs: Differential Maker Genes
PPI: Protein-Protein Interaction
ROC: Receiver Operating Characteristic
AUC: Area Under Curve
GSEA: Gene Set Enrichment Analysis
LASSO: Least Absolute Shrinkage and Selection Operator
SVM-RFE: Support Vector Machine Recursive Feature Elimination
