# Supplementary material for: Identification of Biomarkers Related to Liquid-Liquid Phase Separation for Ulcerative Colitis Based on Single-Cell and Bulk RNA Transcriptome Sequencing Data
Source: Endocr Metab Immune Disord Drug Targets. 2025 Jan 8;25(14):1160–76. doi: 10.2174/0118715303355042241208171133 (PMC12709534; doi:10.2174/0118715303355042241208171133)
Supplement: Supplementary file 1 [file EMIDDT-25-14-1160_SD1.pdf]

Supplementary Material

Identification of Biomarkers Related to Liquid-Liquid Phase Separation for Ulcerative Colitis Based on Single-Cell and Bulk RNA Transcriptome Sequencing Data

Jicheng Lu[1, #], Xu Lu[2, #] and Bin Chen[2, *]

[1]*Department of Oncology, Suzhou Ninth People's Hospital, Suzhou, 215200, China;* [2]*Department of Radiotherapy, Suzhou Ninth People's Hospital, Suzhou, 215200, China*

Table S1. Primers of genes.

**Gene**	**Primer（5'-3'）**
PLA2G2A sense	AAAGGAAGCCGCACTCAGTTATGG
PLA2G2A anti-sense	AGCCTTATCACACTCACACAGTTGAC
GZMK sense	GCGAGAAGTCACTGTTACTGTCCTAAG
GZMK anti-sense	CAGGGTGTAGATTCCAGGCTTTGTG
CD69 sense	TCCTGTCCTGTGTGCTGTAATGAATG
CD69 anti-sense	AGTCCAACCCAGTGTTCCTCTCTAC
HSP90B1 sense	CCAAACGGGCAAGGACATCTCTAC
HSP90B1 anti-sense	CAGGTTCTTCTTCGGGCTCTTCTTC
S100A11 sense	CGGTGCATCGAGTCCCTGATTG
S100A11 anti-sense	GAGGAAGGAGTCATGGCAAGCC
β-actin sense	CACAGAGCCTCGCCTTTGC
β-actin anti-sense	ACCCATGCCCACCATCACG
